# Designing a conceptual model for the formation of unsafe antisocial behaviors in motor vehicle drivers: a grounded theory study

**DOI:** 10.5249/jivr.v14i3.1743

**Published:** 2022-07

**Authors:** Farshad Faghisolouk, Davoud Khorasani-Zavareh, Hamid Soori, Sanaz Sohrabizadeh

**Affiliations:** ^*a*^ Department of Health in Disasters and Emergencies, School of Public Health and Safety, Shahid Beheshti University of Medical Sciences, Tehran, Iran.; ^*b*^ Workplace Health Promotion Research Center (WHPRC), School of Public Health and Safety, Shahid Beheshti Uni-versity of Medical Sciences, Tehran, Iran.; ^*c*^ Safety Promotion and Injury Prevention Research Center, Shahid Beheshti University of Medical Sciences, Tehran, Iran.; ^*d*^ Air Quality and Climate Change Research Center, Shahid Beheshti University of Medical Sciences, Tehran, Iran.

**Keywords:** Conceptual Model, Unsafe Antisocial Behavior, Motor Vehicles, Grounded Theory

## Abstract

**Background:**

Many accidents caused by vehicles are the result of improper driving behavior. Use the vehicle in an antisocial form has led to a phenomenon called driving violence. Antisocial behavior while driving has a potential risk to other road users. This study aims to explain the conceptual framework of the formation of unsafe antisocial behaviors in the road traffic field.

**Methods:**

This qualitative grounded theory study was conducted with exploratory methods. 31 participants were included in this study. Purposeful and theoretical sampling was used in this study. The data collection process was semi-structured interviews. Codes, subcategories, and categories were extracted by the inductive process and analyzed by Corbin and Strauss approach. Trustworthiness criteria were used to assure the quality of the results. The data analysis process continued until there were no new concepts.

**Results:**

A conceptual model was developed to explain the different relationships between the main cat-egories extracted from the study. 10 main categories with 44 subcategories were extracted. Categories include cultural factors, educational factors, rules, economic factors, psychological factors, infrastructure weakness, poor socialization of individuals, violent driving, reduced social welfare and reduced traffic safety.

**Conclusions:**

The people's socialization weakness in society was considered as the core concept in the process of formation of these behaviors. The conceptual model obtained from this study can be used in developing prevention programs and identifying the required interventions Considering the negative consequences caused by this type of behavior, its prevention should be the focus of road traffic policy makers.

## Introduction

The need to drive has increased the number of roads and urban accidents as well as mortality rate; this also is expressed as a problem in the field of public health.^1^ Damages caused by traffic accidents are the main cause of death, disability, hospitalization, and lost expenses in the world.^2^

The use of a vehicle in an antisocial and risky way has created a phenomenon called driving violence, which is a risky driving behavior.^3^ Antisocial behaviors while driving pose a potential risk to other drivers.^4^ There are hundreds of such behaviors that have never been reported and do not lead to law enforcement by the police or authorities.^5^ There are antisocial behaviors in every society and people who have this type of behavior perform a wide range of harmful behaviors such as breaking the law, violating social order, violating the rights of others, aggression, disturbing public order, careless about their own and others safety and being irresponsible.^6^ There is no single definition of antisocial behavior, and these behaviors cover a wide range.^7^ Antisocial behaviors have been studied in different contexts and groups of society, but so far no study has been conducted to investigate these types of behaviors in the context of road traffic, while the evidence shows that these behaviors are associated with traffic accidents.^8^ Studies show that improving the safety of vehicles is only possible if this issue is considered as a social phenomenon and studies focus on the beliefs and attitudes of road users and finding a solution to change that in the future. Since driving is a social activity, the social context in which driving takes place should also be considered.^9^ The tendency to antisocial behaviors in the field of driving may reduce the safety of road users and the safety of the community. Therefore, this study aims to answer the question of what is the formation process of unsafe antisocial behaviors in driving and what are the consequences of these behaviors.

## Methods 

**Study Design**

This is a qualitative study that uses the grounded theory method to examine the subject of the study in detail and finally designed a conceptual model for the formation of unsafe antisocial behaviors in motor vehicle drivers in Iran. This approach was chosen because the experience of the study sample is important to achieve the objectives of the study.^10^ Participants in qualitative research have common experiences in the field of the phenomenon.^11^

**Study Setting**

This study was conducted in Tehran. Iran is one of the deadliest countries in the world in terms of traffic accidents.^12^ The study setting includes traffic police organization, road and urban planning department, emergency department, transport and traffic department, road and road transport organization, taxi organization, road research center, safety promotion, and injury prevention research centers and healthcare centers.

**Participants**

The sampling method was based on the grounded theory approach and was done in two stages.^13^ First, the sampling method was used purposefully to maximize the diversity of participants. In the second stage, the theory sampling method was used which is based on the emergence and saturation of concepts, categories, and subcategories to develop the theory.^13^ Inclusion criteria for participants were having the ability and willingness to conduct interviews and having a valid driver's license and two years of driving experience. Participants in the study were 31 people, including traffic specialists, researchers, and users with specialties in epidemiology, health and emergency, injury prevention and safety promotion, traffic police staff, transport and traffic engineers, psychiatrist, sociologists, psychologists, emergency medicine specialist and motor vehicle drivers.

**Data Collection**

Unstructured and Semi-structured interviews were used to collect data.^14^ findings of one interview determined the questions of the next interview.^15^ The interviews took place between June and September 2019. An example of an interview question was, "Based on your experience, what are the reasons for unsafe antisocial behavior while driving?" Or, "what motivates you to engage in unsafe anti-social behavior while driving?". Data saturation was obtained after 31 interviews. Interviews ranged from 27 to 50 minutes with an average of 31 minutes. A tape recorder and note-taking were used to record the interviews. If the participants were satisfied, the interview was recorded through a tape recorder, otherwise, note-taking is done. 

**Data Analysis**

The Corbin and Strauss method approach was used to analyze the data.^13^ This qualitative research method is very useful when the researcher's goal is to explore a new field or to identify a known field from a new perspective. Data analysis and collecting were performed simultaneously.^16^ Each interview was coded before the next interview. Open, axial and selective coding was used for data analysis. This allowed us to move the results of the theoretical structure towards a conceptual model.^17^ The analysis process was performed using open coding and line-by-line analysis of data to obtain basic concepts. These concepts were then subdivided into different subcategories and categories based on similarities and differences. All codes were checked for accuracy to determine their relationship with other codes.^18^ Then data analysis was continued using axial coding and examining the relationship between categories. Finally, axial coding was formed by constant comparison between categories to extract the core category. Theoretical saturation was achieved when the new data did not provide any new features or dimensions for the categories.^19^

**Trustworthiness**

To reach trustworthiness in this study, four strategies recommended by Schwandt et al, were used:^20^ credibility, confirmability, transferability, dependability. The triangulation method was used to create dependability. In addition to semi-structured interviews, , the researcher uses prolong engagement to achieve credibility.^21^ Peer check will be done through the research team by holding meetings and discussing the data and analysis between the researcher and other experts. In this study, confirmability includes review by observers. To do this, interviews, codes, and categories obtained by several professors specializing in the field of antisocial behavior were examined. Transferability in this study will be done by providing a comprehensive description of the subject, participants, method of data collection, and analysis.^21^ Detailed and accurate note-taking was also used to achieve dependability, which will provide accurate details to other researchers to study or develop similar studies.^21^

## Results

A total of 31 people (including 21 men and 10 women) aged 23 to 67 and an average of 43 years agreed to be interviewed and participated in the study. The demographic characteristics of the interviewees are presented in Table 1. 

**Table 1:** Demographic characteristics of the participants.

Participants information	N (%)
**Gender**	
Male	21(68)
Female	10(32)
**Age**	
20-30	5(16)
31- 40	10(32)
41-50	9(29)
51-60	7(23)
**Participants**	
Experts	19(64)
Drivers	12(36)
**Educational Status**	
Diploma	4(13)
Bachelor	12(38)
Master of science/Medical Doctor	7(23)
PhD/Specialist	8(26)

**Figure 1:**
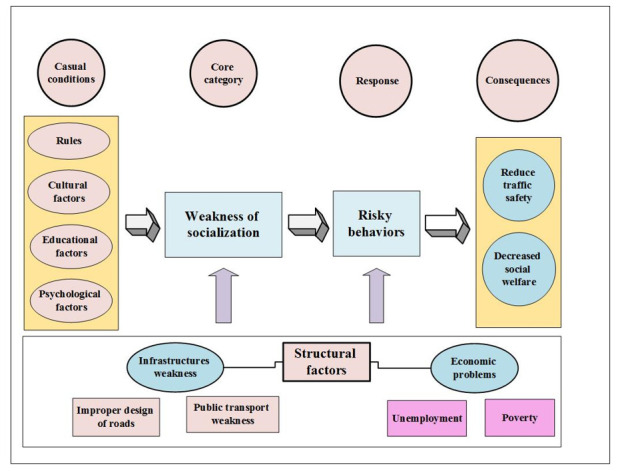
Conceptual model of formation of anti-social unsafe driving behaviors.

10 main categories with 44 subcategories were and 1526 initial codes extracted. Categories include cultural factors, educational factors, psychological factors, rules, infrastructure weakness, economic factors, poor socialization of individuals, risky behavior, decreased Social Welfare, and reduced traffic safety that help us in designing and explaining the conceptual model of antisocial behavior in the area of road traffic (Figure 1). The central categories called poor socialization of individuals which is related to other categories and other drivers' reactions include risky behavior. The consequences of using this strategy include reducing traffic safety and decreased Social Welfare.

**Rules**

According to the participants, antisocial behaviors should be considered in the formulation of regulations, and law enforcers should be supported and guaranteed executively to monitor and enforce these laws. According to them, people who commit these types of behaviors should face more restrictions and be dealt with more severely. Failure to comply with current driving laws will also increase these behaviors in the community. Unsafe antisocial behaviors should be considered in the preparation of driving instructions.

*"These behaviors violate the rights of the people as well as the laws of society" (P9).*

**Cultural Factors**

According to the views of most participants in this study, cultural characteristics of society should be concerned seriously. Controlling these behaviors in the country requires public culture. Identifying cultural weaknesses and increasing and developing the cultural level of society should be included in traffic policies. Participants emphasized technology development in line with cultural needs.

"We discuss cultural delay; i.e., the material aspects work faster than the cultural aspects. Cars and mobile phones work, but thought and philosophy do not work. We live without consciousness. That is, we use a modern product in a non-modern society "(P4).

**Educational Factors**

According to the participants, the provision of public education to the general public by educational centers and mass media, as well as specialized training to drivers of vehicles when obtaining a license and the continuation of these training in the community will increase awareness and knowledge among drivers and thus reducing and preventing unsafe antisocial behaviors. Responsible organizations should also provide the necessary information in this regard to road users.

"We do not provide any education at different levels and in schools. We have to start schools. Our driving schools do not teach civil rights at all. We all have to work together and the radio and television have to provide this information in a special program" (P19).

**Psychological Factors**

According to participants’ experience, no doubt recognizing the diseases and psychological factors affecting antisocial behaviors in driving provides the basis for awareness of the complex dimensions of this social and health issue and can improve intervention strategies. According to interviewees, people with high stress and anxiety are more likely to have accidents due to cognitive problems. Drivers with a score high on abnormal behaviors as a component of mental health are more likely to engage in high-risk driving, and patients with psychiatric disorders generally have higher levels of unsafe antisocial behavior than others.

"Having psychological problems and depression in the community, high anxiety and lack of treatment and addressing these issues" (P23).

**Causal Conditions**

Findings of the study showed that the factors that occurred included cultural factors, educational and training factors, laws, and psychological factors.

**Structural Factors**

Underlying factors include economic factors and infrastructure weakness.

**Economic Factors**

Another condition is economic problems. Unemployment, poverty, and the need to make a living are among the most important economic pressures. Economic pressures on people who engage in unsafe driving behaviors due to poverty and unemployment. Economic pressures within the family change the behavior of drivers about other factors, such as vehicles and the environment.

"One of the most important roots is poverty and unemployment. Most accidents are mostly around poverty and unemployment. The poor and unemployed people do not have proper social status in society". (P3).

**Infrastructure Weakness**

***Poor Public Transport system***

Most participants pointed to the weakness of the public transport structure in the country and its role in the emergence of unsafe antisocial behaviors. Some even said that building highways encouraged people to use private cars. They said that the fleet of public transport should be improved. New and quality buses must enter the transportation system. Because this will reduce people's use of personal vehicles and therefore reduce tension between drivers and ultimately reduce unsafe antisocial behavior. It is necessary to allocate the resources for the construction of roads to the development of public transport.

"In our country, people have to use their vehicles to do their work in the cold of winter and the heat of summer, because there is no public transport system. The crowded buses and difficulties in closing the bus doors are other reasons people have to use their vehicles" (P22).

***Improper road design***

Another reason for such behaviors from the interviewees' point of view was road design. It seems that in the stages of road design and construction, materials and standards related to road safety are not observed, which leads to the formation of accident-prone sections and points in the national road network. Standard roads, convenient intersections, logical red lights, intelligent navigation systems, proper distribution of traffic on the roads, and other measures that can increase the comfort of drivers are effective in reducing high-risk driving. Unauthorized overtaking is usually more on non-standard and crowded roads and non-observance of the right of way occurs more on busy roads. In our country, not enough attention is paid to safety issues and necessary standards in designing road and this tissue leads to some unsafe antisocial behaviors while driving.

"Roads also cause problems and dangerous behaviors. For example, cuts in high-traffic areas, which makes you bored and have to do some behaviors" (P8).

**RESPONSE**

***Risky behavior***

According to the research findings, drivers who have a socialization weakness may engage in risky behaviors in response to other people's antisocial driving behaviors.

"They get aroused quickly, flew off the handle, and commit violent driving. He does not allow anyone to overtake him. Block the road and do everything to annoy others" (P31).

**Consequences**

Consequences of implementing an engagement strategy include reduced social well-being and reduced traffic safety.

***Decreased Social Welfare***

Another consequence is the decreased social welfare in society and the feeling of security in the society and public trust in the transportation system in the country. It is more likely that with the increase of unsafe antisocial behaviors in driving, we will see a decrease in the level of well-being and comfort and an increase in social and psychological harm caused by these behaviors.

"These behaviors affect the peace and privacy of others. Often antisocial behaviors cause annoyance and upset to others and bundle of nerves."

***Reduce traffic safety***

But according to the research findings, it seems that another important consequence will be the increase in traffic accidents and ultimately the death of road users. Injuries and disabilities caused by traffic accidents are increasing in the community and we will see a decrease in traffic safety.

"The unsafe behavior of some drivers distracts other people and drivers and even causes them to have trouble driving and increase the risk of accidents."

## Discussion

The present study used the grounded theory method to discover the process of formation of antisocial behaviors in motor vehicle drivers in Iran. The results showed the interaction between the factors and the different structures for creating these behaviors, which led to the development of a conceptual model. The study showed that the main factor and the central category in the occurrence of these behaviors is the socialization weakness of individuals in society. Socialization weakness leads to irrational individualism in individuals, which is manifested by the display and expression of unsafe anti-social behaviors by the car and the disregard for the public interest. 

According to the findings of the study, one of the effective factors in the formation of unsafe antisocial behaviors while driving is cultural factors. Driving behavior is strongly influenced by the driving culture.^22^ In this regard, a study conducted in Turkey shows that cultural norms in traffic allow drivers to express and commit violence against others while driving.^23^ there is a relationship between cultural factors and dangerous driving manner.^24^ Therefore, it seems that to overcome this problem, it is necessary to develop the traffic culture in the country. Another effective factor in creating these behaviors is educational factors.

 A similar study shows that one of the most important human factors for influencing safe driving is educational issues.^25^ Formal training is positively associated with creating a safe attitude and there is a relationship between driving training on the one hand and safe traffic attitudes and risky driving behaviors on the other hand.^26^ Therefore, it can be said that education and training factors have an important role in this field, and therefore, we should provide general and specialized driving training to people in the community, especially drivers, so that we can reduce anti-social unsafe driving behaviors in them.

Another important factor in creating this type of behavior is psychological factors. Psychological performance is a very important indicator in driving and is considered as an indicator of dangerous behavior while driving.^27^ Understanding the psychological variables that lead to driving behaviors is critical for people working in road safety.^28^ Therefore, we must identify, investigate, and control the impact of these factors on antisocial behaviors among drivers to prevent the occurrence of such behaviors. According to the findings of the study, the weakness of infrastructure is also effective in the formation of these behaviors. High-traffic environments lead to violence, conflict, and intra-individual friction due to underdeveloped infrastructure. The results of a study have shown that congestion and high traffic can lead to violent driving even in countries with strict regulations and good road infrastructure.^23^ Investing in road infrastructure is considered as one of the efforts of countries to improve traffic safety.^29^ Contrary to the results of our study, the results of the some can be stated that the improvement of infrastructure has not played an effective role in reducing overall mortality.^30^ But despite all these studies, it seems that we need to invest in road infrastructure and design principled roads and install appropriate signs on inner and outer city roads.

Laws and regulations in society are also effective in shaping unsafe anti-social behaviors in drivers. The study shows that with the introduction of new driving rules and increased fines for driving under the influence of alcohol in Japan, the country saw a significant reduction in injuries and deaths due to this problem.^31^ Also, the introduction of new rules for crossing and wearing seat belts has shown a negative statistical relationship with mortality and injuries.^29^

According to the findings of the present study, the core and central category in the formation of unsafe antisocial behaviors in motor vehicle drivers are due to socialization weakness. Consistent with our results, a study shows that there is a relationship between dangerous driving and personality traits such as social maladaptation,^32^ and parents play an important role in influencing their children's driving through the process of socialization.^33^ Therefore, since driving in a society is considered as a social activity, the process of socialization of people in the society must be formed properly so that the person commits less unsafe antisocial behaviors while driving.

Economic problems are also effective in creating these behaviors. Consistent with the results of our study antisocial and violent behaviors of adolescents are related to their low socioeconomic status,^34^ and impulsive and antisocial behaviors in children who are under economic pressure are higher than other children. Children in families with high economic pressure are more likely to have symptoms of antisocial behavior.^35^ Another study found that antisocial behaviors are likely more in people who suffered poverty while childhood.^36^ The level of mental health decreases as the economic situation worsens. Poor economic status at the individual and social levels leads to mental disorders. For example, losing a job increases the likelihood of violence. Thus, antisocial behaviors can occur after losing a job. Economic problems cause psychological stress.^37^ One of the strengths of this study is the use of a wide range of participants who have the necessary experience and knowledge regarding this type of behavior. On the other hand, due to the background of anti-social behavior, studies in other places may lead to different results, Therefore, it seems that the existence of economic problems in society can lead to an increase in the occurrence of anti-social behaviors in society.

## Conclusion

The present study extracted various underlying factors in the formation of unsafe antisocial driving behavior and take a deeper look at such behaviors from a social perspective. Examining any type of driving behavior in the field of traffic requires considering the context from which the behavior originates. It is recommended that any study conducted in the field of prevention of drivers' risky behaviors also consider the cultural, social, and economic conditions of the community and based on it, to develop prevention programs. Since these behaviors reduce traffic safety and cause various social harms, so the results of this study can be used to plan and develop the necessary interventions to reduce these behaviors. 
